# An anthropologist’s voice in a veterinarian’s noise: gearing up for new cultural realities

**DOI:** 10.3389/fvets.2023.1202606

**Published:** 2023-08-04

**Authors:** Karmen Šterk, Maja Brložnik

**Affiliations:** ^1^Faculty of Social Sciences, University of Ljubljana, Ljubljana, Slovenia; ^2^Veterinary Faculty, Small Animal Clinic, University of Ljubljana, Ljubljana, Slovenia

**Keywords:** veterinary medicine, small animals, ethnography, social and cultural anthropology, veterinary anthropology, medical anthropology, speciesism, postspeciesism

## Abstract

Over the past three decades, the veterinary profession has faced a cultural shift towards postspeciesism that requires a reassessment of the foundations of the existing distinctions between human and non-human animals proclaimed by the speciesism paradigm, which represents institutionalized discrimination against species and recognizes only the subjectivity of humans. Based on ethnographic observations in anthropological fieldwork and using speciesism/postspeciesism distinction, we aimed to explain the main causes of small animal practitioners’ work-related stress and apply humanistic knowledge to recommend ways to alleviate the negative effects of the work environment. The explanatory model of disease, illness, and sickness, the example of the concept of family, and the circumstances of the feminization of the veterinary profession are discussed to illustrate the divergence between speciesist naturalistic veterinary knowledge and the postspeciesist cultural framework and its consequences. By failing to accommodate the changing values towards animals and by failing to challenge the anthropocentric hierarchy of values, the speciesist rationale of the veterinary profession contributes to many of the problems faced by practicing veterinarians. The incorporation of a modern moral-philosophical mindset towards animals may not even be possible because veterinary science is subject to a paradigm that is irreversibly tied to institutional discrimination against species and defies reflection on veterinary science itself. However, the veterinary profession has a privileged position in establishing an alternative ontological thinking and an alternative conception of “animal life.” Anthropological knowledge was applied to anticipate further intervention of social and cultural sciences in the problems of small animal practitioners. Rather than further diversifying and increasing expectations towards veterinarians by expecting them to acquire additional skills, we propose another practitioner who can support, mediate, and enhance veterinary performance – the cultural anthropologist. With their deep knowledge of cultural differences and social dynamics, they can collaborate with veterinarians to act as a liaison between cultures, paradigms, and species.

## Introduction

Anthropology deals with human affairs in the broadest sense of the term. When the term anthropology is used in this manuscript, it refers to the discipline of social and cultural anthropology, which deals with the totality of the human world. In contrast to physical anthropology, which is concerned only with the physical, biological variations and characteristics of human beings, social and cultural anthropology is concerned with the cultural realities and symbolic environment of human existence (1, 2).

This study presents an anthropological perspective on the veterinary subject and object. Over the past 30 years, the veterinary profession in the Western world has undergone perhaps its greatest paradigm shift. Whether it is called posthumanism, postmodernism, or postspeciesism (3–6), veterinarians today face a gap between the constraints of their professional narrative and the contemporary mindset of the society they serve. The prevailing integrative, inclusive, and compassionate attitudes towards society and nature, informed by the values of sustainability and degrowth, present a whole new set of challenges to veterinary practice that are either not addressed at all or are only referred to in terms of the collision effects they have on veterinarians, such as compassion fatigue, suicide, and substance abuse (7–15).

Speciesism, the paradigm which stands in the core of Modern sciences, is a barely reflected paradigm that denotes a society’s self-evident, unquestioned truths (*doxa* in ancient Greek philosophy) about the hierarchical organization of natural species, according to which humans are considered completely separate from the rest of the animal world and consequently superior in every way. Speciesism is part of the definition of what constitutes the essence of humanity, and always already refers to its antithesis, animality. In fact, virtually all varieties of humanism, whether religious or secular, assume *a priori* that human life has more value and a higher moral standing than animal life. Postspeciesism, on the other hand, is a paradigm that belongs to a broader context of posthumanism and postanthropocentrism and has a completely different epistemology and moral philosophy. It assumes that no single species is in any way better, more valuable, or superior, and that therefore no species should subjugate another. Moreover, it raises the philosophical question of whether a distinction between humans and animals can or even should be made (16–20).

The main issue addressed in this article is the changing attitudes towards animals over the past three decades. Most of these changes can be described by the general concept of the postspeciesism paradigm. In recent decades, animals, especially companion animals, have taken on a completely different status in people’s cultural imagery, value systems, and lifestyles than in the past. Whereas in the past animals were always subordinated to humans and placed at their disposal, today they have acquired not only more, but above all completely different meanings. Animals have bridged the inconmensurable gap between humans and “others,” becoming active members of human intersubjective relations and identity processes, and full members of the family. Institutional veterinary medicine, for the most part, still insists on the old gap, the great divide, the speciesism paradigm that conceives of animals as “others,” as objects and categories defined by market value and utility, though the value and utility in question are solely sentimental (21–29).

We hypothesized that the vast majority of problems facing small animal clinics in Western veterinary practice today are due to the inability of veterinary science to accommodate the nuances of the aforementioned paradigm shift to postspeciesism. Although a commendable number of coping mechanisms have been developed in addition to the existing knowledge of practicing veterinarians (13–15, 30–40), this is still inadequate, and the reason for the inadequacy is precisely qualitative. Not only are animals assigned additional functions to the preexisting ones, but their functions, status, and value have changed radically over the past 30 years.

It seems only logical that institutionalized veterinary knowledge should also follow this paradigm shift, which would imply a reassessment of the very epistemological foundations of the existing distinctions between human and non-human animals, which still largely escape institutional reflection, leaving the “others” on the other side of the divide (41–43).

In this study, we aimed to explain the main causes of work-related stress previously reported in veterinary medicine (7–9, 13, 14) using the speciesism/postspeciesism distinction and to apply anthropological knowledge to recommend ways to reduce and/or mitigate the adverse effects of the work environment on clinicians and their work performance (10, 32).

## Methods

The main methodology used in this study is ethnography. It is the principal qualitative methodology in social and cultural anthropology (44), based on fieldwork and consisting of participant observation, unstructured and semi-structured interviews, shadowing, and haphazard interventions in everyday activities, developing hypotheses about processes and carefully examining unanticipated events (45, 46). The main task of the anthropologist in the field is to get as close as possible to the native point of view of all participants, in our case the veterinarian and the animal caretaker, and to try to grasp the dynamics from the inside, combining this with an outsider’s reflection. By doing so, the anthropologist gains new insights into routines, logics, and inconsistencies that arise from the observed situation itself. As an interpretive science, anthropology then evaluates the observations and contextualizes them with other qualitative or quantitative methods. In retrospect, the result of such an approach is a pilot study that is unique to the particular situation observed but provides new knowledge to the individuals involved, enhances further reflexive evaluations of comparable situations, and enriches the overall understanding of the observed practices (47).

In this study, a cultural anthropologist shadowed a small animal veterinarian at work for 40 weeks (8 weeks per year, 2010–2014), observing the process in the context of clinic emergencies in a private small animal clinic in Slovenia. This allowed observing the main problems small animal veterinarians face in today’s work environment. At the same time, the anthropologist participated in non-technical practical procedures and conducted informal conversations and semi-structured and unstructured interviews with participants. Informed consent was obtained from all participants. After formulating preliminary conclusions from the fieldwork, follow-up interviews were conducted with 14 members of the professional staff (7 veterinarians (5 female, 2 male), 4 veterinary assistants (2 female, 2 male), and 3 female receptionists) and 35 clients (22 female, 13 male). Data collected through participant observation and shadowing in the veterinary clinic were (re)analyzed using the transcribed interviews.

The theoretical contextualization that followed provided the conceptual tools for the subsequent analysis within the existing interpretive framework of relevant social theory (48). Anthropological knowledge was applied at all stages of the research to deepen the understanding of the observed situation and to enhance the subsequent intervention of social and cultural anthropology in the problems affecting the veterinary profession and practitioners of small animal medicine.

## Results

The first and most persistent impression the anthropologist had when approaching the field of small animal emergency practice was that of a variety of fears. It was natural to expect clients to be concerned about the health of the animal they were bringing in for treatment, but that was only one of their concerns. The clients kept casting glances at each other and the receptionist to make sure they were “in the right place.” The veterinarians, on the other hand, tried to appear calm in the waiting room, but behind the scenes their behavior showed clear signs that they were overwhelmed by the whole situation.

Already in the early stages of taking a history and clinical examination, the most common complaint of the veterinarian was about time, “I do not have time for this!” All veterinarians seemed to be in complete agreement on what »this« was, but when asked what they meant by that, they seemed bewildered and were giving vague answers. They all emphasized the emotional burden that animal caretakers placed on them by conveying information that was completely irrelevant to diagnostic procedures, and their hesitation to give consent or make decisions about how to proceed. What clients most wanted to convey with words and by hesitance was the extent and intensity of the animal’s social and emotional value. Most often, they spoke more about an animal’s relationships with family members than about the animals themselves. Such information not only proved irrelevant to the professional assessment, but was counterproductive because of the added psychological and emotional pressure. The veterinarians explained that the main reason they put up with such a situation was the necessary display of empathy that would establish shared signifiers, meanings, moral attitudes, and values of the animal in question. Thus, it was obvious that a veterinarian had to be personally involved in order to convince the animal caretaker that they were speaking the same value-based language. On the other hand, the most common expression of animal caretakers’ resentment toward veterinarians was, “They are only out for money.” On this theme, the clients were unanimous, but when asked how they knew this, the answers were again vague, clichéd, and platitude. When the anthropogist insisted on more detailed reasoning, they complained about the veterinarians’ lack of interest in their animals, which they claimed was “obvious” because they did not engage with their narratives about the animal, and “evident” because they seemed to press the clients for statements that would trigger decisions about costly diagnostic procedures. That “the veterinarians did not care at all about what I told them about the animal” was taken as the ultimate justification for not having confidence in successful treatment.

The misunderstandings were preexistent and manifold, the discrepancy between the discourses was obvious, the skepticism was already present, the value judgments were not based on facts, and the theme of (dis)trust and fatigue and solemn yet very personal involvement permeated the clinic. Even before contacting the veterinarian, the receptionist was frequently asked if the attending veterinarian was ok. When the anthropologist asked what the clients meant by “ok,” they responded that they were interested in the character of the veterinarian; professional competence was of course important, but it was often secondary to “being human.” Another observation immediately stood out: for the anthropologist, it was significant that every client who did not live in a single household referred to the animal as »ours,« even though the client was present alone. For the anthropologist, this was indicative of the animal’s central role in the family and spoke volumes about the role of the animal’s value in the networks of ties binding family members. Moreover, and comparatively significant, such a narrative, even if linguistically incorrect, was not perceived as a mistake by the veterinarians, suggesting that they were aware of the social and emotional surplus value of the animal. However, when the veterinarians spoke to each other, brainstormed, or exchanged ideas about diagnosis and treatment, they consistently referred to the animal either as his/her animal or by symptom or diagnosis, or even referred to the animals by the number of the exam room in which they were located. This is a clear indication that they were trying to create distance between themselves and the animal caretakers, and between their professional persona and the surplus value of the animal, which they were aware of personally but should have no bearing on their considerations. The surplus value was not transferable into technical language and could not be translated into the idiom of the veterinary profession.

To understand the above predicaments, the first section, titled Veterinary Anthropology, discusses the multiple problems affecting veterinarians and the role an anthropologist can play in alleviating the negative effects of the work environment on clinicians and their work performance. The second section, titled Medical Anthropology, explores the various idioms and different epistemological perspectives that come into play in small animal practice. The third and fourth sections, titled Doing Family and Postspeciesism, present the divergence between the postspeciesist moral philosophy of clients and speciesist institutional veterinary medicine.

### Veterinary anthropology: what would you do if it were your animal?

Veterinary medicine’s institutional response to the changing world may be the development of the field of veterinary anthropology that has evolved from the medical humanities whose primary goal is to decenter the role of physician-focused medical knowledge (49). Veterinary anthropology, a philosophical reflection on human–animal relations (50), focuses on the diverse relationships between humans and animals in a sociocultural context. It is an emerging interdisciplinary field that brings together the medical sciences, humanities, and social sciences through the study and care of animals (49–53).

The problems that affect the veterinary profession and practitioners of small animal medicine are manyfold. In addition to their extensive medical knowledge, veterinarians are also expected to possess various non-technical competencies, such as personal traits, values, and skills including ethics, communications, small business management, and other (33, 35, 54). It is tough being a veterinarian. First, veterinarians must serve two masters. They must empathize with the animal and with the animal caretaker, whose interests may conflict (29). Second, the veterinarian is often saddled with incompatible responsibilities, while clients make implausible and divergent demands, expecting the veterinarian to demonstrate supernatural powers in matters of health and appealing to the veterinarian’s compassion by asking an impossible question: what would you do if it were your animal? This is akin to asking a lawyer to first represent both sides in a lawsuit and then to be judge and jury as well (55). Third, there is more pre-diagnostic commentary in veterinary medicine that predicts the official diagnosis and allows for negotiation of diagnosis and treatment than in human clinical practice (39, 56). Suicide rates among veterinarians can be up to four times higher than in the general population, student loans are high, salaries are low, and dealing with problematic clients pales in comparison to the devastating effects of online violence against veterinarians (57–60).

Rather than further diversifying and increasing the expectations of the veterinarian, which would turn work overload into work overkill, we suggest considering another practitioner who can support, mediate, and, in our opinion, significantly enhance veterinary performance – the cultural anthropologist. With their deep knowledge of cultural differences, intercultural competence, symbolic frameworks, and general human affairs, they can work hand-in-hand with a veterinarian to become a liaison between cultures, paradigms, and species.

By adding an anthropologist to the team, rational, technical, and quantitative learning could be complemented by the only methods that produce authentic anthropological knowledge and sensitivity to culture, namely qualitative methods of observation and shadowing. Second, the need for cultural translation would be met. Third, the communication domains constrained by professional interests – whether in healthcare, business, or otherwise – would be broadened and diversified, relieving professionals of more personal obligations and their consequences without depriving the veterinarian of authority and control. Finally, the issue of the emerging demand for interdisciplinarity and the transmission of new academic notions of posthumanism and postspeciesism would be addressed in both institutional veterinary medicine and practice.

The skills used by the anthropologist to achieve this result are difficult to quantify and measure. The soft skills that are increasingly in demand throughout the labor market are unstructured in anthropological work and emerge from the situation itself. They develop from observing, understanding, appreciating, and reflecting on the many nuances of differences in the way people experience their world and any given situation. Communication skills, adaptability, flexibility, non-judgmental empathic understanding, etc. are expected of veterinarians to a degree that they cannot deliver themselves. First, there is the issue of time, then the professional focus, and then the emotional burden. Even when veterinarians are able to provide all of the above, they face a role conflict because their professional role is incompatible with empathy for the emotional, social, and cultural meanings that clients place on their animals. This is most evident when the going gets tough and situations are too emotionally charged to allow for a strictly professional approach, when relevant information is blurred by personal perceptions, and when knowledge-based thinking is influenced by client expectations.

Following a 5 year ethnographic study in a small animal clinic, all participants noted significant changes that we attributed to the presence of an anthropologist. The veterinary lived experience was more professionally fulfilling, communication with clients was less time consuming, problematic clients seemed to lose their edge, and client trust and satisfaction increased significantly as if the aforementioned surplus value of an animal had been consumed by the interaction of the clients and the anthropologist. The active presence of the anthropologist deflected client anxiety, contributed to the experience of treating patients as a team effort, filtered and compartmentalized non-essential data, and in many cases, prevented the potential conflict of roles, attitudes, and paradigms. The code of professional communication was less constrained, colored, and inhibited by other types of verbal and nonverbal communication, which allowed the clinician to focus entirely on professional circumstances. This created an environment in which the veterinarian could remain matter-of-fact without being suspected of lacking empathy for family members.

### Medical anthropology: disease, illness, and sickness

The application of theories and methods from the social sciences and humanities to the natural sciences is neither new nor rare. Medical anthropology draws on social, cultural, biological, and linguistic anthropology to better understand the factors that influence health and well-being (61). In terms of relevance to our study, anthropology as a cultural science has benefited most from adopting the well-established distinction in medical philosophy between disease, illness, and sickness. By this triad, cultural factors were recognized as critical to diagnosis, treatment, and care because they shaped health-related beliefs, behaviors, and values (62–64).

The concept of disease represents the pathological process, structural and functional abnormalities; it is given objective status because it is tangible and measurable. Disease is valued as a central fact of the medical and veterinary universe (65, 66). Illness refers to the perceptions and experiences that individuals have of their condition, which are entirely personal and internal to the person. Sickness pertains to social roles. They are cultural terms for positions negotiated between individuals who deem themselves sick and a society that recognizes them as such (61, 65, 66). The position of being sick is secured by various factors, but the presence of a particular disease is not the most important one. Furthermore, it is a slippery slope to reduce being sick to illness alone (without the diagnosis of disease). On the other hand, having a disease does not imply feeling sick, nor does it necessarily lead to equity in sickness. Chronic diseases are less likely to be equated with sickness than acute ones (65). Sickness is a process of labeling symptoms and expressing their significance both to the person and to their social group. In this process, sickness acquires a definition that is shaped according to certain behavioral patterns, thereby transforming it into a specific cultural form (67). Sickness is an interpretative conclusion given in terms of a particular cultural imagery, which consists of the particular ideas, customs, attitudes, and most fundamental modes of reasoning of a particular society (68).

An example of the disease/illness/sickness model at work can be observed in core veterinary practice of diagnosis and treatment. The pre-diagnostic interview is conducted in terms of the disease and illness, while the technical procedures (laboratory analysis, radiology, biopsy, surgery, etc.) are by definition part of the explanatory model of the disease, and the diagnosis must be presented to the owner in terms of the sickness. Moving from one explanatory model to another implies a translation in which something is always lost and something is always added. In other words, in a clinic, we are always dealing with two native speakers, the veterinarian and the animal caretaker, and with three different linguistic idioms that come with three different explanatory models. Disease is explained in professional terms, combining technical and scientific knowledge that is shaped by individual experience in the workplace. Illness refers to lay, personal, and intimate knowledge, while the narrative of sickness is structured as a discourse that should have common denominators and/or shared meanings, even if the vocabularies and reasonings are different and stem from different epistemologies. This can be illustrated by the well-established anthropological distinction between emic and etic (61, 69). The emic epistemological perspective is a perspective from within, from the perspective of the native language and/or idiom, from the perspective of a native speaker. In our case, it is the perspective of both the caretaker and the practitioner. On the other hand, veterinarians, as native speakers of their own professional idiom, claim their language to be objective, detached, universally applicable, and scientifically valid, i.e., belonging to the etic epistemological perspective. The term etic refers to a perspective from a distance, a perspective from a unified explanatory model that does not include personal and/or subjective experience of a situation to be explained, nor intimate knowledge of the symbolic framework that structures the experience of the second party, the caretaker. Thus, if we are to expect a favorable outcome, the clinician must be able to translate objective knowledge into a subjective symbolic network, and the caretaker must be able to assign meaning to their intimate experience in the context of veterinary medicine. This is easier said than done. To this end, the medical humanities, which have been developing since about the 1970s, were founded to decenter the role of physician-focused medical knowledge and to rely more heavily on the humanities, especially anthropology, linguistics, and literary theory (49). Another attempt to bridge the gap between emic and etic was the introduction of cultural competence in both human (70, 71) and veterinary medicine (33–35, 53). Improved non-technical competencies of practitioners, such as non-discriminatory sensitivity to age, gender, ethnicity, and others, and knowledge of identity processes (awareness of the status an animal has for the person) in taking a history and making a diagnosis, have already been incorporated into small veterinary practice (34, 35). To further address the issue of communication skills (emic/etic distinction) (72, 73), the need to understand the social and psychological reasons for having a companion animal (74) was acknowledged and the prerequisites for mediation in ethical and religious dilemmas regarding vaccinations, invasive procedures, euthanasia, and suspected animal abuse were endorsed (36, 37, 57, 75).

While previous attempts to address the issue of communication skills have undoubtedly contributed to smoother processes and better collaboration between veterinarians and animal caretakers, these efforts have only scratched the surface of the incompatibility of the three discourses (and the emic/etic dyad) that are present at every stage of small animal practice. The existing doctrines applied in veterinary medicine are inadequate due to the widening divergence of the three discourses (disease, illness, sickness) in veterinary medicine, which has occurred in recent decades as a result of tectonic shifts in culture and society towards the objects of veterinary medicine, non-human animals. This growing divergence can be placed in the broader context of changing attitudes towards nature in general and animals in particular.

### Doing family in 21st century

One of the quotidian battlegrounds of the animal–human distinction, and one that practicing veterinarians are constantly confronted with, is the idea of family. In Eurocentric and anthropocentric humanism, the idea of family is associated, if not exclusively with humans, at least with some kind of biological, natural relations and with monospeciesism. However, more than a century of anthropological tradition testifies that, from a cross-cultural perspective, the idea of family and associated kinship terminologies are not cultural labels for natural ties, but rather models for recognizing the cultural significance and social role that one has in relation to another (76). The anthropocentric use of the term family to denote a biologically based entity of humans does not do justice to the diversity and complexity of social (and emotional) forms encountered in the 21st century. In fact, it never did.

Sociological and anthropological studies have already acknowledged the new social reality of so-called hybrid families (also called millennial families, transspecies families, posthuman families, multispecies families, and furry families) (77–81) in which biology is replaced by care, consanguinity by commitment, and function by empathy. To paraphrase the famous feminist dictum that emancipated gender from biology (82), it is not “being” that constitutes gender, it is “doing gender.” Like gender, then, the family is not a naturally given static condition, but a performative behavior of caring, commitment, and empathy. This new reality may be frowned upon, besmirched as sentimental or antropomorphic, and evoke cynical attitudes, but its existence and the turmoil it is causing in veterinary medicine cannot be denied. By failing to keep pace with cultural changes and by not challenging the violent anthropocentric hierarchy of values that always places humans above animals, the speciesist moral philosophy of the veterinary profession contributes to many of the burning issues reported by practicing veterinarians (especially in small animal practice): role strain and overload (9, 13, 37) and occupational health risks such as depression, burnout, and emotional fatigue (14, 83).

Finally, there is the ever-present pressure of the paradigm that “real doctors treat more than one species” (84). The latter takes on a whole new, widespread meaning as pets become part of the human family, with the full legitimacy and emotional endorsement, previously reserved for human relatives, funeral rites included. The increasing use of family surnames on memorial plaques in pet cemeteries is a clear indication of their fixed familial role, literally set in stone (85).

In addition to the pressures of treating family members, whose beneficial influence on mental health has long been recognized (86–88), the veterinarian should approach animal welfare and health from three perspectives: an objective, technical, and professional (speciesist) one; a dedicated, passionate, and empathetic (postspeciesist) one; and an intermediate one that excludes opportunistic and self-serving attitudes of the animal caretaker, renounces the status of ultimate arbiter in animal matters, and takes the animal’s perspective as a point of departure. And what does this mean for veterinary practice? What is called for here is not anthropomorphism and smart sentimentality, but a new ethic of the veterinary profession that is not based on supposed identification with animals, but on respect, appreciation, esteem, and dignity for the otherness of animals.

Furthermore, there are several institutional and linguistic forces at work around and along the animal–human boundary that are anything but subtle in constraining the determination of animality and animal identity according to anthropocentric norms and ideals. First, there is a philosophical dimension that defines identity and hierarchical status through essentialism (attributing language, awareness of death, and reason to humans alone), thus producing forms of exclusion and hierarchization (16). Then there is a pervasive biomedical approach to living organisms with its own taxonomies. Speciesism, which has been at work since before the invention of species, is not a science derived from the observed biological fact that human animals differ from non-human animals. Rather, it is a taxonomic system that produces structural violence, a mere set of prejudices about differences reaffirmed by a moral-philosophical paradigm and shaped into a cultural-cognitive taxonomic system called science (89).

Even if we disregard the unexamined presuppositions of the profession mentioned above, there are seemingly neutral linguistic terms (i.e., quasi-neutral taxonomic models) that convey the same biases as science, reinforce stereotypes, and deepen the differences between one human group and another, and between one species and all others. An interesting example can be found in the Slovenian language, which has different terms for the same biological conditions or events in human and non-human animals (e.g., birth, pregnancy, feeding, death). This clearly defines who is to be empathized with, who is to be cared for, and with whom a family is to be formed. The analogous boundary between subjectivity and objective existence is drawn in the English language by the use of the relative pronouns “who” and “which.” The exclusionary boundary between “who” and “which” refers to the definition of human offspring as children and non-human offspring as puppies, kittens, lambs, kids, piglets, calves, foals, etc. Many people believe that such a distinction is no longer necessary. Nor should the veterinary profession, which is most involved in animal–human relationships, continue to make such distinctions if it is to maintain its authority, respectability, and educability.

One of the unintended but obvious historical contingencies of postspeciesist change is the feminization of the veterinary profession (51, 90). In France, for example, women represent about 55.6% of all veterinarians and 76.5% of practitioners under the age of 30 (91). The situation is similar in the United States and the United Kingdom (57).

To bridge the gap between the demands of the profession and the moral philosophy of the society it serves, the model of the practicing veterinarian has been adorned with female gender role attributes. In contrast to the rationality and controlled, business-oriented mentality attributed to the male gender, where technical skills are paramount, the so-called feminine attitudes of sympathy, compassion, empathy, the capacity for care, and concern and kind-heartedness are increasingly in demand (51, 92).

It may seem paradoxical, but in the broader context of cultural change, it is quite logical that the category of veterinary personnel most affected by the incompatible expectations of the speciesist profession on the one hand, and the increasingly prevalent postspeciesist moral philosophy towards animals on the other, are young female employees (10, 11, 14, 59). Younger women have reported depression, compassion fatigue, burnout, and suicidal ideation more frequently than older women or their male counterparts (38, 57, 59), which may largely reflect the changes in perceptions of the animal in social organization and cultural taxonomies. This may be due to the speciesist framework embedded in the socialization process of older generations, as well as the male gender role that still demands control, detachment, objectivity, and rationality. Hence, professional distance comes naturally to them, which also provides a solid frame of reference for professional self-confidence. The latter is reflected in the established correlation between mental health on the one hand and age and gender on the other (10).

The younger generation of women, born into the postspeciesist moral-philosophical framework in which animals are part of the family, does not have the luxury of institutionalized speciesism. They have a higher prevalence of poor mental health (11, 59) and more commonly find themselves in a “betwixt and between” position characterized by the prevalent and continually reported work-related stressors arising from the “caring-killing paradox,” (convenience) euthanasia, adverse events in elective surgery, animal suffering, and others (13, 37–39, 93, 94). To add insult to injury, they are paid less than their older and/or male colleagues (60, 91, 95).

### Postspeciesism: dogs are people too

Speciesism, in the extreme, is institutionalized discrimination between different species that favors humans and recognizes only the subjectivity of humans, while other species are considered inferior and mere objects that serve the purposes of humanity (3). There is supposedly an unbridgeable gap between human and non-human animals, and the grounds for this distinction, and hence discrimination, are both arbitrary and increasingly inadequate: the number of legs, the villosity of the skin, or the end of the *os sacrum* (17). Numerous other arbitrary criteria for establishing hierarchical distinctions between animals in speciesist frameworks exist, and they all classify animals according to the principle of utilization. The core of speciesism is evident in the “pet, pest, profit” classification used in veterinary medicine (96), which does not do justice neither to animals nor the contemporary social mindset.

The speciesist epistemology of living things, i.e., objects, inherent in veterinary medicine contrasts with the modern non-anthropocentric and postspeciesist cultural epistemology, which is the core issue leading to the multitude of problems in veterinary practice today. Practitioners and their clients literally no longer speak the same language. The languages used today are no longer compatible with the advent of postspeciesism, lacking common denominators, shared frames of reference, moral philosophies, and value systems. This predicament is somewhat analogous to the triad of illness (a personal perspective, i.e., how it feels to be ill), disease (a professional perspective, i.e., how health care professionals define, recognize, predict, and handle disease entities), and sickness (a social perspective, i.e., how a person’s social role is defined or altered by social norms and institutions). However, the comparison is limited because the aforementioned rift between speciesism and postspeciesism elevates these three perspectives to a new level. While these perspectives, i.e., disease, illness, and sickness, focus on different phenomena and entities, involve different kinds of knowledge, and require different actions by health professionals (66), postspeciesism requires novel epistemologies and ontologies of human and non-human animals (16).

The naturalistic human–animal distinction of the 21st century, based on anthropocentric norms and ideals, can and should no longer be sustained. Several political, ethical, and ontological reasons are provided for this argument by the continued rise and widespread presence of animal rights activists, social movements for climate justice, biodiversity, and sustainability, as well as by the most influential philosophers of the contemporary era (6, 17, 19). All of them challenge the existing hegemonic scientific, social, and political structures, anthropocentrism, and chauvinism that underpin modern institutions (20, 25, 26, 97). Contrary to the widespread belief that society’s survival and future require unequal valuation of humans and animals (55), posthumanism and postspeciesism reject the moral neutrality of the idea of progress, for it is precisely the idea of progress combined with anthropocentric criteria that has led to scientific and technological developments in the breeding, slaughtering, and utilization of animals to improve human welfare. The idea of progress, combined with modern humanistic ontologies that exclude animals from moral consideration, has led to the unprecedented subjugation of animals. Animals are cruelly slaughtered, unconscionably abused, and kept in unimaginable living conditions. This has pushed many species to the brink of extinction and destroyed ecosystems, biodiversity, and cultural diversity. Structural violence towards animals and nature, which is at the core of speciesism, has become one of the most important philosophical, moral, political, and cultural issues of our time (17, 20).

## Discussion

This is the first study to contextualize the current prevailing issues of small animal medicine practice in broader context of veterinary science epistemology.

The veterinary profession has not followed the changing moral and value-based attitudes toward animals in the modern Western world, resulting in veterinarians facing numerous difficulties that they have never experienced in their professional environment. We argue that this has also been impossible in part because veterinary science is subject to an underlying paradigm that seems to defy reflection on veterinary science itself – the paradigm of speciesism. However, the veterinary profession is in a privileged position to take the lead in laying the groundwork for an alternative ontological thinking and an alternative notion of “animal life” that departs from the reductive accounts of animality in the history of modern science. It is not clear what kind of thinking will emerge once reliance on these categories is abandoned. What is certain, however, is that any genuine encounter with what we call animals will only occur if we move beyond the current speciesism paradigm. Transformation is necessary and inevitable.

In the veterinary profession, various coping mechanisms for work-related stress have been employed (13, 15, 30, 37–39), however, virtually no attention has been paid to exploring the root causes of occupational health risks such as emotional fatigue, burnout, and social media victimization. The building blocks, compassion expectations, the “caring-killing paradox,” and burnout, have been present for some time, but recently we have seen an exponential increase in associated stress. This can only be mediated by a deeper understanding of the cultural forces at work and the emerging social realities. Veterinary medicine is attempting to deal with this by incorporating the human sciences into veterinary curricula (27, 51), but it remains questionable whether existing scientific, economic, and legal institutions can be reshaped to accommodate the coming social and cultural realities created by the postspeciesism paradigm. The acquisition of humanistic knowledge is undoubtedly informative, but it could also lead to additional work and role overload for veterinarians, which are already cited as high stressors.

In the meantime, small animal practices could consider adding an anthropologist to their staff as an intermediary. This would serve a dual purpose: an anthropologist could mediate between professionals and animal caretakers by translating cultural predicaments into veterinary practice and professional language into a contemporary cultural mindset. Furthermore, an anthropologist working in the clinical setting would provide a novel opportunity to acquire additional knowledge that veterinarians need in a contemporary postspeciesist society. In short, the presence of an anthropologist would enrich the core quantitative naturalistic knowledge taught by institutional veterinary medicine with humanistic qualitative methodological approaches, such as shadowing that would enable veterinarians to see themselves through the reflexive lens of anthropological attention (50).

It is not difficult for anthropology to view recent human history (and the sciences in general) as a series of divides overcome, for it was precisely anthropology that first viewed differences (between human groups) as something non-essentialized, non-ontological, and neutral. It considers the various valuations of differences primarily as a consequence of a series of violent interventions of *quasi*-scientific taxonomic models into observed reality, resulting in a variety of imperialisms inherent in the Eurocentric worldview. Whether economic, political, scientific, or merely terminological and epistemological imperialisms, they all led to a moral evaluation of differences and assigned these differences a place in hierarchical taxonomic models. Most of this was done to expose differences previously attributed to nature as arbitrary, sociohistorical constructs. First, 100 years ago, there was the issue of race, which was redefined as a social and cultural category, not a natural one. Race is something that belongs to the cultural taxonomic system of assigning differences between human groups based on skin color (89). Then, 60 years ago, there was the issue of human biological sex, which was denaturalized and assigned the term gender through the intervention of cross-cultural data. At the same time, the quasi-natural Darwinian sexual binarism was abolished, bringing about the emancipation and equality of the sexes and, later, the demise of heteronormativity. In retrospect, taxonomic models of nature can be seen as always arbitrary, cultural, and culturally specific explanatory models. To put it bluntly, “natural facts” are concepts, not things in themselves, so they are not really discovered, but invented, essentialized, and defined through the use of a particular pre-existing symbolic paradigm. When discussing “natural facts,” it is their symbolic framework that should always already be considered (67).

With this background, anthropology appears to be best equipped to challenge the next divide that is central to defining humans as a unique category: the distinction between humans and animals, or between human and non-human animals. Anthropology is well-suited to address the interplay, paradoxes, and problems that arise from the conflict between relatively rigid veterinary conceptions of animals and the changing attitudes toward animals, particularly companion animals, in contemporary Western societies. Over the past 30 years, the role of animals has undergone a significant transformation. Dogs and cats have become family members (28), living in our homes, sleeping in our beds, having birthday parties and receiving Christmas gifts (35). The animals are now genuinely loved and mourned (98), and are buried with memorial plaque inscriptions (85, 99). These memorial plaque inscriptions reflect this shift in attitudes, with fewer using the word “pet” and more using human names (85). Moreover, institutional practices previously reserved for humans, such as hospice care, open-heart surgery, organ transplants, and others, have been introduced into animal health care.

Legislation is also changing: laws against animal cruelty are becoming stricter, charities that advocate for animal rights are increasing in number, and penalties for animal abuse, cruelty, and abandonment are increasing. The amount of money families spend on a companion animal is increasing, as is the market value of pet products. Social media has also played a role in amplifying these shifts in attitudes, with algorithms connecting people based on their interests and values, and allowing groups with specific interests to better organize and achieve greater reach and influence. On the other hand, the anonymity provided by social media has also led to an increase in bullying of veterinarians, which has become one of the most commonly cited causes of stress and even suicide (58).

From being a functional necessity for food and safety, animals have in many ways become a model for our humanity. We use them to learn compassion, empathy, fearlessness, grace, devotion, and character. They live with us as part of the family; we identify with them; they are key to our lifestyle, consumer and even political choices; and they promote social integration, psychological well-being, and learning abilities (28, 100–102). Today, it is not at all uncommon for a large portion of the Western population to identify and empathize more with an animal than with a fellow human being. Affirmations of self-image and moral worth, identity politics, leisure activities, choice of residence, and decisions about personal spending are increasingly associated with and dependent on animals. Attitudes and moral philosophies towards animals that decades ago were reserved for eccentrics, romantics, aristocrats, and outcasts are becoming mainstream. This is reflected in the numbers: at most ages Americans are more likely to have pets than children (103).

The “great divide” of modern knowledge, resulting from the modern ontological separation between the human and the non-human and the corresponding division between the natural sciences and the humanities (29, 83), is becoming increasingly apparent and excessively redundant. Veterinarians are encouraged to be socially sensitive, and many small animal clinics have introduced grief counseling for professionals (15, 104) and animal loss support groups that provide socially sanctioned mourning (31). Social sensitivity has definitely been expanded on the whole, but this is largely due to the tendency to optimize processes to maximize profits, and, to a lesser extent, to create a less stressful, less competitive, and less perfectionistic work environment for practitioners.

However, the role of the veterinarian in the changing attitudes towards non-human animals in human society, especially companion animals, has become so diverse and multifaceted that further steps must be taken to address the growing number of issues related to veterinary practice. These include bridging the widening gap between the emic positions of the profession and animal caretakers; dealing with the multiple roles that animals have come to represent to people and that are reflected in people’s expectations of veterinarians; overcoming the mutually exclusive paradigms of naturalistically defined pets and humanistically recognized full-fledged companions; and confronting the dichotomous requirement to be simultaneously objective, technical, calm, cool, and collected (pertaining to the profession) and committed, passionate, and empathetic (pertaining to the culture).

Overall, the veterinary profession should develop a sensitivity to the many indicators of how and why the cultural definition of animals is radically changing. Sociology and anthropology can provide context for these changing attitudes, translate these attitudes into client expectations of veterinarians, contribute to improved marketing strategies, and more. The social sciences and humanities can illuminate how a (post)modern individual can be addressed as a set of self-affirming identity politics consisting of fighting the climate crisis and carbon emissions, collecting plastic from the oceans and collecting newspapers for charity, donating to environmental organizations, and protesting against heteronormativity. Having an animal as a family member is like the cherry on top and leads one to recognize oneself as a non-discriminatory, self-actualized project belonging to a better socio-economic class and endowed with noble posthumanistic values of closeness to nature (25, 97). Indeed, it is animals, especially those at the extreme ends of the cultural spectrum, companion animals, and wild animals, that have become symbols of the norms, morals, and ideals of 21st century humanity: loyalty, fidelity, attachment, bonding, and unconditional love on the one hand, and courage, independence, and perseverance on the other. Whether anthropocentric, display oriented, empathy-driven, or simply fashionable, animals have become a new role model for humanity.

And what does this mean for veterinary practice? The physical condition and behavior of animals and animal–human interactions can provide insight into relationships in the household, predict the framework of interpersonal dynamics, and detect potential family (dys)function (105). The recognition of dual-income-no-kids (DINK) families (106) and/or the concept of conspicuous consumption (107) helps to determine the exclusivity of emotional attachment to an animal; attitudes towards neutering, euthanasia, and hospitalization; and profiles of consumer behavior and market segments. The introduction of critical social theory, which reveals the late 20th century as a period of disenchantment and disillusionment with fellow human beings (108), provides a frame of reference for understanding shifting empathies, compassion, and loyalties. The recognition of non-binary personal identities can increase personal sensitivity in pre-diagnostic interviews. The recognition of an animal as a formative factor deeply embedded in posthumanistic identity processes may shed light on people’s preferences for noninvasive diagnostic procedures and treatments. Sensitivity to the social niches that animals occupy in a household can provide invaluable information about their living environment (109), which is essential for history taking. Insights into ancient classical literature, artistic performances, and references to animals in the dramas of Shakespeare, Ibsen, and Chekhov as signifiers of the inclusion, permeability, and interchangeability of humans and animals (110) suggest the recognition that humanity has always been incomplete without a connection to the non-human animal. Further on, institution of veterinary medicine that accepts animals as family members allows for instant rapport, replaces burdensome personal moral and emotional engagement with institutional commitment, and thus minimizes the occurrence of large-scale misunderstandings and mistrust on the part of postspeciesist clientele. Finally, the presence of an anthropologist in small animal practice would enrich the learning culture, which has suffered greatly from institutional veterinary medicine not speaking the idiom of postspeciesism that clients speak, and education is regrettably becoming an irreversible, one-way process.

This anthropological study has shown that some of the main problems of veterinary practice in the Western world are related to the incompatible paradigms of institutional veterinary medicine on the one hand and the cultural context on the other. To bridge the gap between the two, we propose the inclusion of an anthropologist on the professional staff who could provide a veterinarian with culturally sensitive information and convey professional knowledge in the language of contemporary society. Further studies are needed to confirm the results of this study, preferably on larger samples and with mixed methods, i.e., qualitative and quantitative (111, 112). In addition, multi-sited research (113) could examine social and cultural phenomena in different and diverse locations and from different and diverse perspectives, adding a comparative dimension to the study. The theories of ecofeminism (114, 115) and critical animal studies (116, 117) have not been included, but could provide a complementary framework for analyzing the shortcomings of the veterinary profession in confrontation with posthumanist moral philosophy.

## Data availability statement

The original contributions presented in the study are included in the article/supplementary material, further inquiries can be directed to the corresponding author.

## Ethics statement

The requirement of ethical approval was waived by Ethical Committee at the Faculty of Arts, University of Ljubljana for the studies involving humans because the study included data obtained by observation and participation in public activities in which no sensitive personal data was collected; publicly accessible data or prior existing deidentified data; and semi-structured interviews for which the researchers obtained written informed consent. The studies were conducted in accordance with local legislation and institutional requirements. No potentially identifiable images or data were included in this article.

## Author contributions

KŠ: study concepts/study design and data acquisition. KŠ and MB: data analysis/interpretation, manuscript drafting, manuscript revision for important intellectual content, approval of the final submitted manuscript, and manuscript editing. All authors contributed to the article and approved the submitted version.

## Conflict of interest

The authors declare that the research was conducted in the absence of any commercial or financial relationships that could be construed as a potential conflict of interest.

## Publisher’s note

All claims expressed in this article are solely those of the authors and do not necessarily represent those of their affiliated organizations, or those of the publisher, the editors and the reviewers. Any product that may be evaluated in this article, or claim that may be made by its manufacturer, is not guaranteed or endorsed by the publisher.
